# Screening Blood and Vitreous for Biomarkers Associated with Proliferative Diabetic Retinopathy

**DOI:** 10.3390/diagnostics15111344

**Published:** 2025-05-27

**Authors:** Stephen Richard, Rawan Kharouba, Jawad Abu-dbai, Oksana Gagarin, Assaf Kratz, Basel Obied, Alon Zahavi, Nitza Goldenberg-Cohen

**Affiliations:** 1 The Krieger Eye Research Laboratory, Bruce and Ruth Faculty of Medicine, Technion—Israel Institute of Technology, Haifa 3525433, Israel; steverit11@gmail.com; 2 Department of Ophthalmology, Bnai Zion Medical Center, Haifa 3339419, Israel; rawan.kharouba26@gmail.com (R.K.); jawad.abu-dbai@b-zion.org.il (J.A.-d.); oksana.gagarin@b-zion.org.il (O.G.); basel.obied01@gmail.com (B.O.); 3 Department of Ophthalmology, Soroka University Medical Center, Beer Sheva 84101, Israel; 4 Faculty of Health Sciences, Ben Gurion University of the Negev, Beer Sheva 8410501, Israel; 5 Department of Ophthalmology and Laboratory of Eye Research, Felsenstein Medical Research Center, Rabin Medical Center—Beilinson Hospital, Petach Tikva 49100, Israel; alonzahavi@gmail.com; 6 Faculty of Medicine, Tel Aviv University, Tel Aviv 69978, Israel

**Keywords:** vitreous, gene expression, mRNA, biomarkers, neovascularization, retinopathy, proliferative diabetic retinopathy

## Abstract

**Background/Objectives**: Uncontrolled or long-standing diabetes can lead to proliferative diabetic retinopathy (PDR), a condition that significantly impairs vision. A subset of patients does not respond adequately to conventional therapies, such as intravitreal injections of anti-vascular endothelial growth factor (VEGF) or laser treatment. This study aims to identify potential biomarkers for alternative treatment pathways in the vitreous and blood of patients with severe PDR. **Methods**: Vitreous samples were collected from PDR patients (*n* = 3) undergoing vitrectomy for vitreous hemorrhage and from control patients (*n* = 9) undergoing ocular surgery for epiretinal membrane or macular holes. Blood samples were collected from a separate group of PDR patients (*n* = 13) and non-diabetic control patients without retinopathy (*n* = 13). Medical histories were obtained. Two-stage real-time quantitative polymerase chain reaction (qPCR) was used to evaluate mRNA expression levels of genes potentially implicated in PDR, including *HIF2A*, *PAI-1*, *TIE1*, *TIE2*, *ANGPT2*, and *VEGFA*. Molecular and statistical analyses were performed to compare PDR and control vitreous and blood samples. **Results**: The PDR vitrectomy group included two females and one male, aged 71–77 years (mean 74 years). All participants had undergone pan-retinal photocoagulation and two had received anti-VEGF injections before vitrectomy. These participants had elevated HbA1c levels. Targeted vitreous gene analysis revealed varying levels of increased expression of all genes examined as compared to the control group. A trend for increased median expression was demonstrated for all examined genes: *HIF2A* by 1.44-fold (PDR = 2.50, control = 1.74, *p* = 0.21), *PAI-1* by 1.56-fold (PDR = 3.00, control = 1.93, *p* = 0.37), *TIE1* by 1.36-fold (PDR = 2.33, control = 1.72, *p* = 0.66), *TIE2* by 2.06-fold (PDR = 2.81, control = 1.36, *p* = 0.51), *ANGPT2* by 2.93-fold (PDR = 6.32, control = 2.16, *p* = 0.1), and *VEGFA* by 3.53-fold (PDR = 3.51, control = 0.99, *p* = 0.08). PDR blood sample analysis as compared to controls showed a trend for increased expression of *VEGFA* by 1.2-fold (PDR = 0.88, control = 0.74, *p* = 0.57), whereas the other examined genes showed a trend of reduced expression; *HIF2A* decreased by 0.50-fold (PDR = 0.38, control = 0.75, *p* = 0.07), *PAI* by 0.51-fold (PDR = 0.35, control = 0.69, *p* = 0.09), *TIE-1* by 0.79-fold (PDR = 0.79, control = 1.00, *p* = 0.54), *TIE-2* by 0.70-fold (PDR = 0.58, control = 0.82, *p* = 0.34), and *ANGPT2* by 0.45-fold (PDR = 0.51, control = 1.15, *p* = 0.11). **Conclusions**: Vitreous sample analysis revealed a trend of increased mRNA expression of *ANGPT2* and *VEGFA* in patients with PDR. Blood sample analysis did not show a significant increase of *VEGFA* mRNA expression but a decreased trend of *HIF2A*, *PAI-1*, and *ANGPT2* mRNA expression. These trends warrant validation in a larger cohort to explore alternative pathways for targeted treatment.

## 1. Introduction

Diabetes is among the most prevalent diseases in developed countries [1]. Prolonged high glucose levels in the blood (hyperglycemia) can lead to retinal blood vessel damage and reduced oxygen supply to the retina. The resulting oxidative stress in the retina triggers the release of angiogenic factors, which leads to neovascularization [2]. Small retinal blood vessel pathology due to diabetes with or without vascular proliferation (PDR, proliferative, NPDR, non-proliferative diabetic retinopathy, respectively) is the most common complication in diabetic patients and one of the main causes of adult blindness in the developed world [3]. Retinal neovascularization in PDR may result in fluid extravasation from the blood vessels and retinal edema, retinal hemorrhages, vitreous hemorrhages, and fibrovascular membrane development, which can be complicated by retinal detachments, leading to visual morbidity and irreversible visual loss [1,4,5]. Currently, the main treatment is pan-retinal photocoagulation (PRP) laser treatment or intravitreal injection of anti-vascular endothelial growth factors (VEGFs). Laser treatment destroys retinal tissue leading to reduced oxygen consumption, thereby minimizing VEGF secretion. However, it does not directly influence vascular endothelial cell pericytes [6]. Intravitreal anti-VEGF injections directly inhibit VEGF, mitigating its vascular proliferative effects. Despite the combination of these two treatments, some patients are resistant to treatment or do not improve sufficiently [5].

There is growing evidence that PDR has a genetic predisposition. Several studies were conducted to identify other possible proliferation factors or genes that may serve as alternative targets for treatment. Gene-expression profiling showed changes in fibrovascular membranes and extracellular matrix-related molecules of PDR patients [2]. The concentrations of tissue plasminogen activator (t-PA), proliferative factor- plasminogen activator inhibitor (PAI), and VEGF in the vitreous of PDR patients were shown to be significantly higher as compared to controls. The t-PA and PAI expressions were highly correlated with the increased VEGF expression [7]. PAI-1, expressed in endothelial cells and pericytes, was found to be dysregulated in vitreous samples from patients that did not respond well to treatment [8]. In addition, a mouse model demonstrated that the inhibition of PAI-1 prevented the development of abnormal retinal blood vessels in the ischemic retina [9].

Previous studies have not fully elucidated the molecular changes underlying human retinal pathologies, particularly in PDR. Furthermore, molecular biomarkers identified in blood and other tissues do not always accurately reflect the primary retinal changes. Given its close proximity to the retina, the vitreous presents a valuable medium for investigating these molecular changes. This study aims to identify dysregulated biomarker genes in vitreous and blood samples from patients with PDR, which may be associated with resistance to current therapies and could serve as potential targets for novel molecular treatments.

## 2. Materials and Methods

The study cohort included a study group of PDR patients and a control group of non-diabetic patients, scheduled for vitrectomy due to vitreous hemorrhage, retinal detachment, macular hole, or epiretinal membrane peeling. Additionally, a separate group of PDR patients and non-diabetic control patients without retinopathy was recruited for blood sample collection.

Following Institutional Review Board approval and informed consent, medical history was reviewed for systemic diseases, diabetes control, HbA1C levels, and target organs complications (neuropathy, nephropathy, and retinopathy). A comprehensive ocular history was documented, encompassing clinical examinations and treatment history, including details of prior laser therapy and/or anti-VEGF injections. The recorded data included the patient’s response to treatment, the number of treatments received, the type of interventions, and their frequency.

Immediately after general or topical anesthesia, 2–3 mL of undiluted vitreous samples were obtained via standard three-port pars plana vitrectomy using a 23-gauge vitreous cutter with a 5 mL syringe. During acquisition, the infusion was set to air to prevent dilution. Samples were aliquoted to sterile tubes and stored immediately in a −80 °C storage freezer pending laboratory analysis.

Peripheral venous blood samples (1.5 mL) were collected in an EDTA-coated tube, stored at room temperature, and processed within 2–3 h.

The vitreous samples collected were processed for RNA extraction. Next, 350 µL of TRIzol reagent (Invitrogen, Carlsbad, CA, USA) was added to 350 µL of each vitreous sample, mixed, and incubated in ice for 30 min. After incubation, the mixture was homogenized using a hand-held battery-operated homogenizer, then pulled ‘up-and-down mixing’ using an 18G needle and syringe to break long nucleic acid secondary structures, and underwent vortexing. Chloroform (70 µL) was added to each mixture and incubated at room temperature for 3 min. After incubation, the mixture underwent centrifugation at 13,500 rpm for 20 min at 4 °C. The upper aqueous phase was moved to a fresh tube and 250 µL of isopropanol was added. This mixture was incubated overnight at −20 °C, then centrifuged at 13,500 rpm for 20 min at 4 °C. The pellet was gently washed using filtered 75% ethanol. The mixture was then centrifuged at 13,500 rpm for 10 min at 4 °C. The supernatant was discarded and the RNA pellet was dissolved in 20 µL nuclease-free water. RNA concentration was measured using NANODROP (2000 UV-VIS Spectrophotometer, Thermo Scientific, Waltham, MA, USA). The ratio of absorbance at 260 nm/280 nm was checked to be within the range of 1.8–2. Following extraction, RNA samples were processed for cDNA synthesis.

Peripheral blood samples were processed for RNA extraction using the QIAamp RNA Blood Mini Kit (Qiagen, Hilden, Germany) according to the manufacturer’s instructions. In brief, one volume of whole blood was mixed with five volumes of erythrocyte lysis buffer and incubated on ice for 15 min, then centrifuged at 200 rpm for 10 min at 4 °C. The pelleted leukocytes were lysed with RLT buffer. The lysate was homogenized by passing it through a QIAshreder spin column. The homogenized lysate was combined with an equal volume of 70% ethanol, loaded onto a RNeasy spin column, and centrifuged at 13,000 rpm for 30 s. On-column DNase digestion was performed to eliminate DNA contamination. The RNA bound to the spin column membrane was washed with RW1 and RPE buffers and then eluted with nuclease-free water. RNA concentration was measured using NANODROP (2000 UV-VIS Spectrophotometer, Thermo Scientific, Waltham, MA, USA). RNA samples were processed for cDNA synthesis.

Approximately 1000 ng of the extracted RNA from vitreous or blood samples was utilized for each cDNA synthesis reaction. The reaction mixture was prepared with the following components: 1000 ng of RNA, UltraPure water added to the RNA to achieve a final volume of 15 µL, 4 µL of 5X buffer, and 1 µL of QuantaBio-qScript MMLV Reverse Transcriptase (1 U/µL). The cDNA synthesis was performed using the following thermal cycler program: an initial cycle at 22 °C for 5 min, followed by a cycle at 42 °C for 30 min, then a cycle at 85 °C for 5 min, and a final hold at 4 °C. After the reaction, the obtained cDNA was diluted 10-fold and about 20 ng of cDNA was taken for each well in the quantitative polymerase chain reaction.

Primers were designed for *HIF2A*, *PAI-1*, *TIE1*, *TIE2*, *ANGPT2*, *VEGFA*, and *ACTB* genes in humans. For efficient amplification, primers (between 18 bp and 25 bp long) were designed so that the amplicon was between 90 and 120 bp long. The melting temperature for each primer was kept to a minimum of 60 °C and a maximum of 64 °C, utilizing a Tm calculator (https://www.thermofisher.com (accessed on 23 April 2023)). The list of primers is presented in Table 1. All primers were examined for efficiency using the standard curve method. Human cDNA samples with high expression levels of the target genes were selected for determining the primer efficiencies.

Two-stage real-time quantitative polymerase chain reaction (PCR; QuantStudio 3; Applied Biosystems, Foster City, CA, USA) was used to evaluate levels of mRNA expression of genes possibly implicated in proliferative diabetic retinopathy; *HIF2A*, *PAI-1*, *TIE1*, *TIE2*, *ANGPT2*, and *VEGFA*. Human beta Actin (*ACTB*) was used to normalize cDNA input levels. The primers are listed in Table 1. Reactions were performed in a 20 µL volume containing 4 µL cDNA, 0.5 µM each of forward and reverse primers, and buffer included in the master mix (Fast SYBR Green I; Applied Biosystems, USA). Duplicate reactions were performed for each gene to minimize individual tube variability and an average was taken for each time point. Threshold cycle efficiency corrections were calculated, and melting curves were obtained using cDNA for each gene PCR assay. PCR cycling conditions consisted of an initial denaturation step of 95 °C for 20 s followed by 40 cycles of 3 s of denaturation at 95 °C and 30 s of annealing and extension at 60 °C. The conditions used for the melt curve stage were 15 s of denaturation at 95 °C and 60 s of extension at 60 °C followed by 15 s of denaturation at 95 °C. The results were quantified using a comparative threshold cycle (Ct) method, also known as the 2^−ΔΔCt^ method, where ΔΔCt = ΔCt (PDR) − ΔCt (non-diabetic control). Molecular analysis was performed comparing the PDR and non-diabetic control in vitreous and blood samples.

Statistical analysis of gene expression levels was performed by comparing the groups. The statistical significance was set as a *p*-value ≤ 0.05 by a non-parametric test—Mann–Whitney U test—usingGraphPad Prism, version 10.4.1 (GraphPad Software, a Dotmatics company, Boston, MA, USA).

## 3. Results

### 3.1. Vitreous Gene Expression Analysis

**Figure 1:**
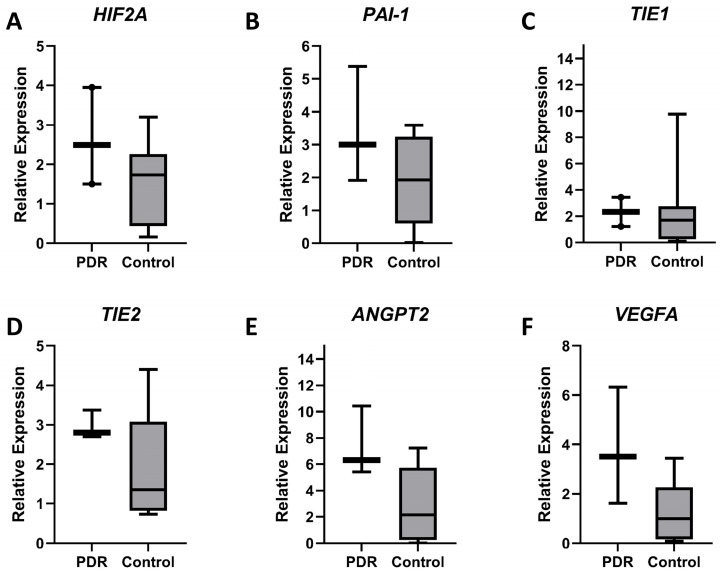
Targeted mRNA expression analysis of vitreous samples by qPCR. The expression levels of the genes (**A**) *HIF2A*, (**B**) *PAI-1*, (**C**) *TIE1*, (**D**) *TIE2*, (**E**) *ANGPT2*, and (**F**) *VEGFA* were assessed. The y-axis represents the relative expression, shown as median with interquartile range. Statistical comparisons between groups were performed using the Mann–Whitney U non-parametric test. Statistical significance was set to *p* < 0.05.

Twelve vitreous samples were analyzed, including three from patients with PDR and nine from non-diabetic controls. Gene expression levels were assessed using qPCR. *ANGPT2* and *VEGFA* median expression showed an upward trend, being elevated in PDR vitreous compared to controls, reaching close to statistical significance, with *ANGPT2* showing a 2.93-fold increase (PDR = 6.32, control = 2.16, *p* = 0.1) and *VEGFA* a 3.53-fold increase (PDR = 3.51, control = 0.99, *p* = 0.08). Although not close to statistical significance, the median expression levels of all other analyzed genes were also higher in PDR vitreous. *HIF2A*, *PAI-1*, *TIE2,* and *TIE2* expression showed an upward trend, with *HIF2A* increasing by 1.44-fold (PDR = 2.50, control = 1.74, *p* = 0.21), *PAI-1* by 1.56-fold (PDR = 3.00, control = 1.93, *p* = 0.37), *TIE1* by 1.36-fold (PDR = 2.33, control = 1.72, *p* = 0.67), and *TIE2* by 2.06-fold (PDR = 2.81, control = 1.36, *p* = 0.52) in PDR vitreous compared to controls (Figure 1).

### 3.2. Blood Gene Expression Analysis

**Figure 2:**
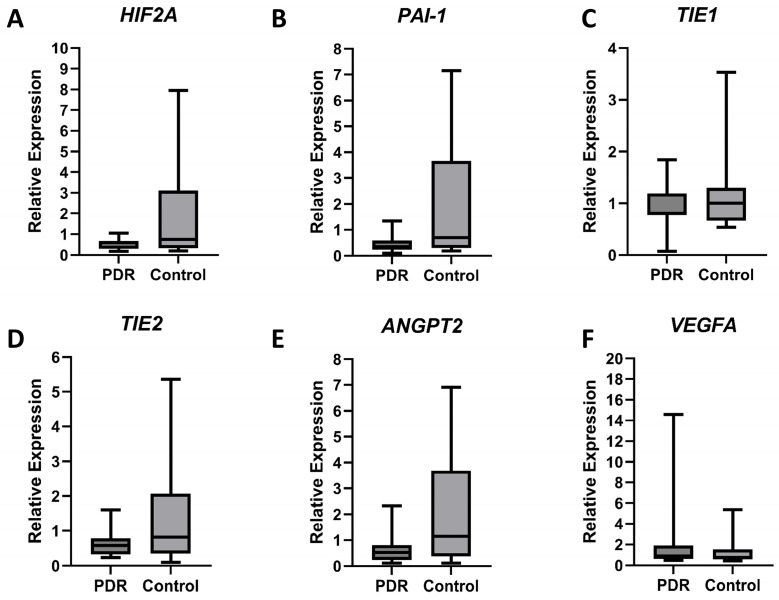
Targeted mRNA expression analysis of blood samples by qPCR. The expression levels of the genes (**A**) *HIF2A*, (**B**) *PAI-1*, (**C**) *TIE1*, (**D**) *TIE2*, (**E**) *ANGPT2*, and (**F**) *VEGFA* were assessed. The y-axis represents the relative expression, shown as median with interquartile range. Statistical comparisons between groups were performed using the Mann–Whitney U non-parametric test. Statistical significance was set to *p* < 0.05.

Gene expression was assessed by qPCR in blood samples drawn from patients with PDR (*n* = 13) and non-diabetic controls (*n* = 13) (Figure 2). Median relative expression of *HIF2A*, *PAI-1*, and *ANGPT2* genes showed a downward trend in PDR compared to controls, reaching close to statistical significance, with *HIF2A* decreasing by 0.50-fold (PDR = 0.38, control = 0.75, *p* = 0.07), *PAI-1* by 0.51-fold (PDR = 0.35, control = 0.70, *p* = 0.09), and *ANGPT2* by 0.45-fold (PDR = 0.52, control = 1.15, *p* = 0.1). Although not close to statistical significance, most other analyzed genes also exhibited reduced expression in PDR patients, except for *VEGFA*, which showed a 1.19-fold increase (PDR = 0.88, control = 0.74, *p* = 0.57). In contrast, *TIE1* and *TIE2* displayed a downward trend, with *TIE1* decreasing by 0.79-fold (PDR = 0.79, control = 1.00, *p* = 0.54) and *TIE2* by 0.70-fold (PDR = 0.58, control = 0.82, *p* = 0.34) in PDR patients as compared to controls (Figure 2).

## 4. Discussion

In this study, we examined the gene expression of novel targets in the vitreous and blood of patients with PDR. Our analysis of vitreous samples demonstrated an upward trend of *ANGPT2* and *VEGFA* mRNA expression in PDR patients reaching close to statistical significance. In contrast, blood sample analysis did not reveal a significant increase in *VEGFA* mRNA expression but showed a marked reduction in *HIF2A*, *PAI-1*, and *ANGPT2* mRNA levels.

The posterior segment of the eye is filled with vitreous humor, a delicate transparent gel composed of 99% water, proteins, and polysaccharides [10]. Positioned in close proximity to the retina, the vitreous can undergo changes that mirror the pathophysiological processes occurring in retinal tissue [11]. Notably, significant alterations have been observed in the vitreous of diabetic retinopathy (DR) patients compared to non-diabetic individuals [10]. Samples obtained via pars plana vitrectomy from patients with PDR reveal altered levels of structural and functional bioactive molecules, including pro-angiogenic, pro-inflammatory, and neurotrophic mediators, bioactive lipids, non-coding RNAs, and extracellular vesicles [10].

Anatomically, the vitreous is contiguous with the retina, lens, ciliary body, and retinal blood vessels, allowing bioactive compounds and cells from these regions to infiltrate during pathological conditions. Though predominantly composed of water, proteins, and polysaccharides, the vitreous contains a small number of cells concentrated in the cortex [12]. Hyalocytes constitute 90% of these cells, with fibroblasts accounting for the remaining 10%. Cells such as laminocytes, macrophages, and microglial cells are localized near the vitreoretinal interface, the inner limiting membrane (ILM), and collagenous adhesions, where they may play roles in adhesion and enzymatic collagen breakdown [13,14,15,16]. These cellular constituents, whether indigenous or infiltrated during pathology, can provide insights into retinal molecular dynamics, particularly in PDR [13].

Our quantitative PCR analysis identified the presence of all examined mRNAs in vitreous samples. The mRNA level changes observed are consistent with known alterations in PDR [8,9,13,17,18,19,20,21]. Notably, median *VEGFA* mRNA expression increased by 3.53-fold in PDR vitreous compared to controls (Figure 1), corroborating reported increases in VEGFA protein levels in PDR vitreous [22,23]. VEGFA, a key angiogenic driver, is instrumental in the pathological neovascularization characteristic of PDR [24].

The *ANGPT2* mRNA expression also showed marked upregulation (2.9-fold, Figure 1). Additionally, although the increase of *PAI-1* (1.56-fold; Figure 1), a regulator of thrombosis and fibrinolysis, and *HIF-2α* (1.44-fold, Figure 1), a driver of the hypoxia pathway, were not close to statistical significance, their upward trends align with established roles of hypoxia, inflammation, and angiogenesis in PDR pathogenesis [8,25]. Hypoxic conditions, mediated by HIF-2α stabilization, drive VEGF expression and neovascularization [25,26]. Increased PAI-1 levels, a serine protease inhibitor, may contribute to impaired fibrinolysis, cell migration, and extracellular matrix remodeling [9,27]. Our data corroborate reported increases in PAI-1 protein levels in PDR vitreous [8]. Similarly, the Ang/Tie pathway, through ANGPT2, facilitates vascular destabilization and enhances VEGFA-mediated neovascularization and inflammation [19,20,28]. Our data demonstrate increased mRNA levels of *ANGPT2*, and *VEGFA* in the vitreous (Figure 1). Notably, the observed upregulation of *ANGPT2* and *VEGFA* mRNA levels aligns with previously reported elevated protein levels of these factors in the vitreous of patients with PDR [23]. This suggests that PDR-induced vascular leakage and neovascularization also affect the vitreous. Importantly, this study highlights the vitreous as a valuable window into retinal pathologies, with observed gene expression changes reflecting retinal disease dynamics.

Additionally, the analysis of blood samples from PDR patients revealed systemic involvement. *VEGFA* expression showed a 1.2-fold increase (*p* = 0.57; Figure 2), suggesting its potential as a non-invasive biomarker for disease progression. Although the increase did not reach statistical significance, this trend supports the growing body of evidence implicating VEGFA in the pathophysiology of PDR [29,30,31]. Previous studies have shown elevated VEGFA protein levels in the vitreous [22,32] and serum [33] of PDR patients, and our findings suggest that VEGFA may also be upregulated in whole blood samples.

In contrast, several other genes implicated in retinal angiogenesis and vascular function, including *HIF2A*, *PAI-1*, and *ANGPT2*, exhibited decreased median expression in PDR blood samples, reaching close to statistical significance (Figure 2). Specifically, *HIF2A* and *PAI-1* levels showed a marked downward trend by 0.50-fold (*p* = 0.07) and 0.51-fold (*p* = 0.09), respectively. *HIF2A* is a key transcription factor involved in the hypoxic response, which is essential for retinal neovascularization in PDR [8]. The reduced expression of *HIF2A* in peripheral blood samples may suggest a dysregulated hypoxic response in PDR patients, potentially reflecting the complex interplay of hypoxia and inflammation in the systemic environment. The observed decrease in *PAI-1* expression in blood samples of PDR patients may be indicative of alterations in the coagulation and fibrinolytic pathways, or consumption, which are important in the pathogenesis of diabetic retinopathy. However, the precise role of PAI-1 in peripheral blood versus ocular tissues in PDR remains unclear, warranting further investigation.

*ANGPT2*, a key regulator of angiogenesis and endothelial cell signaling [18,19,20], exhibited reduced expression in PDR blood samples (Figure 2). TIE1 and TIE2 are receptor tyrosine kinases that govern endothelial cell survival and angiogenesis [18,28], while ANGPT2 is pivotal in mediating endothelial dysfunction and vascular permeability [20]. The reduction in *ANGPT2* expression was not statistically significant yet presented a trend (*p* = 0.1) underscoring its potential role in the systemic vascular dysregulation characteristic of PDR. In contrast, the reductions in *TIE1* and *TIE2* expression did not reach close to statistical significance (*p* > 0.05), indicating that these markers might be less reliable for systemic monitoring of PDR compared to *HIF2A*, *PAI-1*, and *ANGPT2*. The dysregulation of these pathways highlights a complex interplay in the pathophysiology of PDR, warranting further investigation into their contributions.

Despite the preliminary nature of this study and the limited sample size, the statistical significance threshold was set to *p* ≤ 0.05. A more lenient threshold may be considered appropriate in exploratory biomarker research, where the primary goal is to identify promising molecular targets for further study rather than to establish definitive clinical associations. This would reduce the likelihood of Type II errors (false negatives), ensuring that potential biologically meaningful signals—such as upregulation of ANGPT2 and VEGFA in vitreous samples—are not prematurely dismissed. These findings should be interpreted with caution and validated in larger, more powered cohorts. When the *p*-value was calculated using a t-test based on the mean, the results indicated statistical significance (*p* < 0.05). However, given the small sample size, a more accurate non-parametric approach—the Mann–Whitney U test—was employed. This yielded *p*-values above 0.05, indicating a consistent trend despite not reaching conventional significance.

This study represents a novel investigation into mRNA expression in both vitreous and blood samples from PDR patients. Future research should focus on validating these findings in larger cohorts and examining the relationships between gene expression in peripheral blood and ocular tissues. Characterizing biomarkers predictive of severe diabetic retinopathy or resistance to treatment could enable early diagnosis, tailored interventions, and improved patient outcomes. The identification of new genes involved in neovascularization has the potential to expand treatment options, either as standalone targets or in combination with existing anti-VEGF therapies. Early identification of patients at risk for PDR or treatment resistance could pave the way for personalized and more effective treatments, ultimately reducing diabetes-related blindness and improving quality of life.

## 5. Conclusions

This study identifies key molecular pathways involved in PDR that may contribute to locate new targets for treatment, for those patients with treatment resistance and disease progression. The elevated mRNA expression of *ANGPT2* in vitreous samples suggests that this factor may serve as alternative or adjunct therapeutic targets to anti-VEGF treatments. Future studies with larger cohorts are needed to confirm our findings and explore their therapeutic potential.

## Figures and Tables

**Table 1:** List of primers used for qPCR.

*HIF2α_F*	5′ GATGACAGAATCACAGAACTGATTGG 3′
*HIF2α_R*	5′ GCACAAGTTCTGGTGACTCTTG 3′
*PAI-1_F*	5′ AGATTGATGACAAGGGCATGGC 3′
*PAI-1_R*	5′ GAAGATCGCGTCTGTGGTGC 3′
*TIE1_F*	5′ GGAAGAGCAACGGATCCTACT 3′
*TIE1_R*	5′ TAGATGCCGCTCGATGGTGG 3′
*TIE2_F*	5′ ATAGTCCGGAGATGTGAAGCC 3′
*TIE2_R*	5′ GCATTCTCCAGTATCTTCATGGCA 3′
*ANGPT2_F*	5′ TGGAAGCTGGAGGAGGCG 3′
*ANGPT2_R*	5′ TGAAGGGTTACCAAATCCCACT 3′
*VEGFA_F*	5′ ACCTCCACCATGCCAAGTGG 3′
*VEGFA_R*	5′ GTAGCTGCGCTGATAGACATCC 3′
*ACTB_F*	5′ CTCTTCCAGCCTTCCTTCCT 3′
*ACTB_R*	5′ AGCACTGTGTTGGCGTACAG 3′

## Data Availability

The raw data supporting the conclusions of this article will be made available by the authors on request.

## Abbreviations

The following abbreviations are used in this manuscript:

PDR	Proliferative diabetic retinopathy
NS	Not significant
mRNA	Messenger ribonucleic acid
